# Integration of *Vitis vinifera* By-Product Powder into Sponge Cake to Create Innovative Functional Food with Improved Physical, Quality and Sensory Characteristics

**DOI:** 10.3390/foods15040671

**Published:** 2026-02-12

**Authors:** Otilia Cristina Murariu, Nadezhda Golubkina, Gianluca Caruso

**Affiliations:** 1 Department of Food Technologies, ‘Ion Ionescu de la Brad’ Iasi University of Life Sciences, 700490 Iasi, Romania; 2 Federal Scientific Vegetable Center, Selectsionnaya 14, VNIISSOK, Odintsovo District, 143072 Moscow, Russia; 3 Department of Agricultural Sciences, University of Naples Federico II, 80055 Naples, Italy; gcaruso@unina.it

**Keywords:** pastry products, grape pomace, textural properties, color, polyphenols, antioxidant activity

## Abstract

The present research aimed at assessing the effects of the integration of *V. vinifera* pomace powder, resulting from wine production, into the manufacturing of a sustainable pastry product. Comparisons were made between 9 treatments derived from the factorial combination of 3 tescovine powders (obtained from 3 different manufacturers) added to sponge cakes and 3 concentrations (3%, 5% and 10%), plus a control without tescovine powder addition. Textural, quality and color parameters, as well as bioactive compounds, antioxidant activity and sensory features were determined. The highest porosity and elasto-plastic coefficient were observed for the untreated control, and it was found that the values reduced as grape pomace incorporation increased. The rising application of tescovine powder enhanced the textural parameters of sponge cakes compared to the untreated control, with the highest average level observed with Natur Tanya integration. Resilience, cohesiveness and elasticity decreased as the concentration of grape pomace rose to 10%. Gummosity and chewability displayed increasing trends from the untreated control to the 10% tescovine powder addition. Dry matter content, acidity, sugars, mineral substances and ash increased from the control to the highest grape pomace integration, contrary to the pH trend. With rising tescovine powder incorporation, the sponge cake color components L* and b* decreased compared to the untreated control, whereas the component a* showed increasing values in the cake crumb but decreasing values in the crust. The highest antioxidant activity and compound levels were recorded with the 10% Fiber Foods grape by-product addition. Most sensory characteristics showed a decreasing trend with increasing tescovine powder integration from 3% to 10% in the pastries analyzed. This study demonstrated the benefits of adding tescovine by-product to sponge cakes to manufacture innovative foods with high nutritional value.

## 1. Introduction

The bakery industry has been increasingly developing, and innovative solutions are being sought for creating functional flour-based products that are beneficial to human health because they are rich in bioactive compounds, minerals, proteins and fiber [1,2]. Within the mentioned sector, pastries are among the most widely consumed flour-based foods across different social and economic categories [3]. In this respect, research has been focusing on the integration of different plant ingredients, particularly through the valorization of by-products such as grape pomace, into the manufacturing of bakery products [4]. In particular, the present study refers to the use of tescovine waste for healthily enriching sponge cake, which belongs to the category of products leavened by the incorporation of air and is widely appreciated by consumers around the world [5]. The functional food produced represents an attractive proposal of innovative items with enhanced physical, nutritional, color and sensory attributes for consumers [6]. Moreover, the antioxidant compounds provided by these additions are protected in the gastrointestinal tract by the gluten elastic network developed during the kneading process, which confers healthier properties to the final functional food [7].

*Vitis vinifera* L. is spread worldwide, covering 185,000 hectares and 62.26 million hectoliters of wine production in 2024, according to the International Organization of Vine and Wine (OIV). The winemaking process results in significant amounts of waste, which in the present study are represented by tescovine (pomace), accounting for about 20–30% of the initial grape weight [8].

The red grape pomace mentioned above has stood out over time for its high content of bioactive compounds, making it a recommended functional ingredient in the context of a healthy lifestyle. The main constituents identified in this by-product include dietary fibers [9], polyphenolic compounds, anthocyanin pigments and minerals [10], which contribute to the regulation of intestinal transit and the maintenance of a healthy gut microbiota, with beneficial effects on the digestive system [11,12], as well as protection against chronic diseases, including cancer, neurodegeneration and cardiovascular pathologies [13]. The chemical composition of tescovine varies as a function of some factors, such as pedoclimatic conditions (temperature, precipitation) and the technology used in the winemaking process [14]. Previous studies have highlighted significant differences between 5 types of tescovines in terms of dietary fiber, proteins, polyphenols, flavonoids and antioxidant capacity [15]. Grape pomace was initially used as animal feed [16], but its high concentration of minerals [17], proteins [18], lipids [19], soluble and insoluble dietary fibers [20], and minerals [21] has led to interest in integrating it as a by-product for the development of innovative foods [22]. However, concerns regarding the safety of by-product addition to food have arisen because of possible physicochemical, microbiological and toxicological contamination [23].

Novel functional foods obtained by manufacturing products added with waste or beneficial substance [24,25,26,27] are defined as “natural or processed foods that contain effective and non-toxic amounts of bioactive compounds, which provide clinically proven health benefits documented through the use of specific biomarkers for the prevention of human aging, the treatment of chronic diseases, or their symptoms.” [28].

Based on the bioactive grape pomace characteristics, numerous studies have been conducted regarding the integration of this by-product into various food categories. The latter strategy has significantly improved the properties, referring to fiber, protein, lipids, anthocyanin content, and antioxidant capacity in the finished products, such as pastry [29], pasta-type bakery [30], bread-type [31], and breadstick-type [32].

This study proposes a sustainable way to integrate food waste into pastry manufacturing, thus preventing environmental and soil pollution [33], as supplying tescovine to soil leads to a drastic decrease in oxygen and groundwater quality because of its low pH and tannin content [34].

The purpose of the present research was assessing the effects of incorporating grape pomace powders, by-products of wine making, derived from 3 different manufacturers, at three different concentrations (3%, 5% and 10%), into sponge cakes on their physical, quality, color, antioxidant and sensory properties.

## 2. Materials and Methods

### 2.1. Research Location and Experimental Protocol

**Figure 1:**
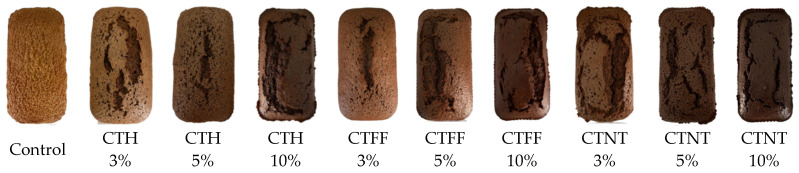
Experimental treatments.

Research was carried out at the University for Life Sciences “Ion Ionescu de la Brad” in Iasi (Romania), in 2025. The experimental protocol included 9 treatments obtained factorially combining 3 tescovine skin powders of red grape (*Vitis vinifera* L.) obtained from 3 different manufacturers (Herbal Sana—CTH, Fiber Foods—CTFF, and Natur Tanya—CTNT), added to sponge cakes, and 3 concentrations (3%, 5% and 10%), plus an untreated control, for a total of 10 treatments with 3 replicates (Figure 1).

The effects of the mentioned experimental treatments on physico-chemical (porosity, elasto-plastic coefficient, F max compression, resilience, cohesiveness, elasticity, gummosity and chewability, immediately after cake manufacturing and 7 days later), quality (dry matter, acidity, pH, immediately after cake production and 7 days later, and sugars, proteins, fats, mineral substances, and ash), antioxidant (activity, vitamin C, polyphenols, flavonoids, anthocyanins) and sensory properties of the produced sponge cakes were determined.

The powder of red grape skins obtained from three different manufacturers through different processing technologies (Table 1), which may affect the final functional food properties, was used to create innovative sponge cakes providing health benefits. The maximum addition of 10% tescovine powder was chosen because, based on our trial (not published), a higher grape pomace integration percentage negatively affected the sensory characteristics as well as product fineness and, during chewing, a pronounced graininess was perceived.

The average percentages of retention in the finished products, of the substances integrated into the sponge cakes, referring to the 3 levels (3%, 5% and 10%) of grape pomace additions per each manufacturer, have been calculated as the ratio between the theoretical and the measured content in the final pastries. The chemical composition of the tescovine powders incorporated into the cakes has been used for the mentioned theoretical calculation (Table 1).

### 2.2. Grape Pomace Powder and Cake Preparation

The three grape pomace powders added to the sponge cakes were manufactured by Herbal Sana, Natur Tanya and Fiber Food, stored in dry and cool conditions at the Department of Food Technologies of the University for Life Sciences “Ion Ionescu de la Brad” in Iasi (Romania).

The sponge cakes are based on ingredients, such as flour, sugar, eggs and other auxiliary ingredients [35], which make them suitable for becoming functional food upon tescovine powder addition. The latter solid material was used with a small granulation to achieve a successful outcome in the manufacturing process, especially during the homogenization and molding stage, to have also a positive impact on the color and texture parameters.

### 2.3. Technological Process

The ingredients used to obtain the experimental samples were initially subjected to preparation processes such as coding, washing, conditioning by weighing, sieving and separation. A 000-type cake flour was weighed and shifted three consecutive times, both to remove any impurities and to give it aeration. The eggs (about 70 g/piece) were washed in the special egg sink and then separated into coded bowls. Extra-fine white sugar was weighed according to the recipe. Baking powder (10 g/plicle), vanilla sugar (10 g/plicle) and vanilla essence (38 mL/vial) were added, the latter two auxiliary materials needed to provide flavor to the experimental samples.

The mentioned formula ingredients refer to the control sample, whereas the experimental treatments were created by calculating the red grape skin powder concentrations of 3, 5 and 10% out of the total product weight.

The additive material was calculated as a percentage of the total ingredient weight to ensure the comparability of formulations and to avoid excessive disruption of the gluten structure. This approach is commonly used in functional bakery product manufacturing where bioactive ingredients are added as enrichment components rather than flour substitutes, especially when their technological role is nutritional and antioxidant rather than structural. The mentioned formulation strategy ensures dough processability, fermentation performance, and product integrity in all experimental treatments.

The grape pomace powder, stored in a dry and cool place, was coded according to the producer (Herbal Sana—H, Fiber Foods—FF, Natur Tanya—NT) and then weighed in compliance with the 3 planned concentrations.

The flour and red grape skin powder were uniformly mixed before being added to the product, to ensure its homogeneous distribution and, consequently, the uniform final product color and texture. The manufacturing process of the ten experimental samples started with the preparation of the foam consisting of egg whites, sugar, vanilla essence and sugar, and yolk beaten with salt and water beforehand and added to the foam formed from the first ingredients [36] in a planetary mixer (model Plutone 10, Sirman S.p.A. Curtarolo, Padua, Italy).

The resulting product was slowly added with the combined flour, tescovine powder (except for the untreated control) and baking powder, and the mixture (320 g) was poured into coded disposable molds. The baking process took 45 min at 175 °C [37], after which the cakes were taken out of the oven and kept at ambient temperature for 1 hour [6], then packaged in plastic containers with lids and stored at 2–4 °C; a countersample of each treatment was frozen, in case of need for some analyses.

### 2.4. Determination of Textural Properties

The texture of the experimental treatments was measured with the Mark 10 Plus texturometer (Mark-10 Corporation, Copiague, NY, USA), according to Wee et al. method [38]. A TA10 cylindrical probe with 12.7 mm diameter, 35 mm height and 0.05 N resolution was used for the compression, at the displacement velocity of 180 mm per minute. After inserting the probe into the experimental samples, 2 types of diagrams were recorded: Fd, force-displacement, and Ft, force-time; a specific software, (GraphPad Prism, version 9), was used for their interpretation. The following physical measurements were made just after manufacturing the final product and 7 days later: porosity, elasto-plastic coefficient (k), maximum compression strength, resilience, cohesiveness, elasticity, gummosity, chewability. The height, width and thickness of the samples were determined using a Mitutoyo 500-196-30 digital caliper (Mitutoyo, Kanagawa, Japan) with 0–300 mm measuring range, ±0.02 mm accuracy and 0.01 mm resolution.

### 2.5. Determination of Quality Parameters, Water Activity and Color Components

Dry matter was determined at 130 °C for 1 hour in the Biobase laboratory forced air-drying oven (Biobase^®^, Jinan, China) [39].

The determination of the cake sample pH and titratable acidity were made according to literature indications [39,40]. In particular, pH was measured by a handheld pH meter (Hanna Instruments), which was initially calibrated with buffer solutions at pH 7 and 4. An aqueous extract consisting of the sample and warm distilled water was prepared and, after 30 min, the pH was measured by inserting the 2 electrodes into the obtained extract with 3 repetitions.

Sugars, fiber, proteins, lipids and mineral substances were determined in compliance with AOAC International [39].

The inorganic residue was measured by calcining the sample at 500 °C for 3 h in a Snol 8.2/1100 calcining furnace, followed by a 30-min repetition to check the constant weight [39].

Water activity of cake samples stored for 7 days at 2–4 °C was determined as previously reported [7].

The color characteristics of the products were analyzed 24 h after baking end using a Chroma Meter CR-410 (Konica Minolta INC., Chiyoda-Ku, Tokyo, Japan) on the CIELAB scale. The related components L* (lightness), a* (red to green) and b* (yellow to blue) were analyzed [41], both externally and internally, on the whole samples and their sections. Prior to the measurements, a white calibration was made using the related supplied plate and, after calibration, the instrument accuracy was validated by measuring reference standards with known color values.

### 2.6. Determination of Antioxidant Activity

The antioxidant activity was measured using the DPPH (2,2-diphenyl-1-picrylhydrazyl) procedure. A DPPH stock solution (25 mg 100 mL^−1^ methanol), diluted by 1:10 with methanol, and then a TROLOX stock solution (25 mg 50 mL^−1^ methanol) were prepared. The readings were taken at 515 nm absorbance [42].

### 2.7. Determination of Vitamin C Content

The vitamin C content (mg 100 g^−1^) was determined by titrimetric method with 2.6 dichlorophenolindophenol [43]. The reagent 2.6 DI was prepared 24 h before use, kept cold and filtered. The point indicating the titration end was represented by the appearance of a permanent pink coloration [44].

### 2.8. Determination of Polyphenol Content

The determination of total polyphenol content was carried out by the Folin–Ciocâlteu colorimetric method, using a 0.20 mL diluted sample (1:10), 15.8 mL distilled water and 1 mL Folin–Ciocâlteu reagent. The resulting mixture was stirred for 10 min, after which 3 mL of 20% sodium carbonate was integrated, and the obtained solution was incubated at room temperature for 60 min [45]. The readings were performed by a spectrophotometer at the 765 nm wavelength [46], using gallic acid (GA) as calibration curve standard [47]. The values obtained are expressed as mg GA g^−1^ dry matter.

### 2.9. Determination of Total Flavonoid Content

A 0.250 mL volume of dilute extract (1:10) with 1.25 mL distilled water and 0.075 mL 5% sodium nitrate was used to determine the total flavonoid content. The obtained solution was left to react for 5 min, then was integrated with 0.15 mL of 10% aluminum chloride and the resulting mixture was left to react for 6 min. Finally, 0.5 mL of 1 M sodium hydroxide and 0.775 mL of water were added, after which the readings were taken at 510 nm wavelength against a blank. The total flavonoid content was measured by the catechin standard curve [48].

### 2.10. Determination of Anthocyanin Content

The anthocyanin content was measured by the differential pH method. Diluted samples (1:10) were placed (200 µL of sample and 800 µL of pH 1.0/pH 4.5 buffer solution) in the cuvette of the Janway UV-Vis spectrophotometer (Fisher Scientific, Loughborough, Leicestershire, UK) and left standing for 15 min. The readings were taken at 520 and 700 nm absorbance [49].

### 2.11. Sensory Analyses

The sensory characteristics of the 10 experimental treatments, with random 3-digit numerical codes from V1 to V10 in the questionnaire, different between sessions to prevent product recognition, were determined by QDA (Quantitative Descriptive Analysis) as previously reported [50], in the sensory analysis laboratory of the University for Life Sciences “Ion Ionescu de la Brad” in Iasi (Romania). A questionnaire was applied to a panel made of 20 people (10 women and 10 men) with experience in the field, aged between 30 and 45, non-smokers, without known cases of food allergies, reducing the effects of order (by individual randomization) and sensory fatigue, with the aim of obtaining a detailed sensory profile of innovative sponge cake products. This study was reviewed and approved by the Ethics Committee for Research in Human Beings of the Department of Food Technologies, Faculty of Agriculture, of the mentioned University. In this respect, each evaluator gave a written informed consent to participate in the activity and was informed about the right of withdrawing from it at any time, in compliance with the European Union Guidelines of Ethics and Food-Related Research [51] The cake samples, represented by 2 cm thick and 25 g weighed slices, were randomly placed on platters [29]. The overall preference and acceptability of the products were evaluated using a 9-point hedonic test. Each sensory characteristic analyzed (general aspect, purchase intention, overall acceptability, fruity flavor, bitter taste, sweet taste, salty taste, wine-like taste, elasticity, crumb aeration, crumb volume, crumb color, crust color, uniformity degree of addition) was evaluated on a scale of 1 to 9, where scores between 1 and 4 signify non-compliance, 5 to 7 signify improvement, and 8 to 9 signify compliance. Each evaluation criterion was explained in a sheet accompanying the questionnaire, detailing all the characteristics associated with each score category. Scores were not totaled for each sample to classify it into a category of conformity or acceptability, but a separate question was inserted for each sample regarding overall acceptability, where a score of 1 signified ‘Not at all acceptable’ and 9 signified ‘Very acceptable’.

### 2.12. Statistical Processing

The data statistical processing was made by one-way analysis of variance, performing the mean separation through the Duncan’s multiple range test at *p* < 0.05, using the SPSS software v. 30.

## 3. Results and Discussion

### 3.1. Physical Properties

Regarding the physical properties measured just after the product manufacturing (Table 2), the top porosity level was recorded in the untreated control, with gradual reduction upon increasing the grape pomace integration; seven days later, the mentioned parameter showed a greater drop in the control cake, compared to the lowest tescovine concentration. Similar trends were recorded for the elasto-plastic coefficient, but the untreated control had the highest value both after the cake production and a week later.

The untreated control showed a soft texture either in the just manufactured cake or seven days later (Table 2), typical of aerated pastries, with a hardness of 2.2 N, whereas the increasing integration of tescovine powder enhanced the textural parameters. Indeed, sample hardness attained the highest values at the 10% concentration, within each grape pomace type, i.e., 15.5 N with the Natur Tanya, compared to 8.9 N of Herbal Sana powder and 9.6 of Fiber Foods. The Natur Tanya powder had a higher grain size compared to 300–400 microns of the other two powders. The 3% grape by-product addition did not generally differ statistically from the untreated control for all the textural parameters, suggesting that the mentioned concentration is optimal for texture.

Resilience decreased upon rising tescovine powder addition, indicating a reduced ability to return to the original shape after compression (Table 3). However, the latter parameter had an opposite trend seven days after the manufacturing completion. The cohesiveness showed a similar trend as the resilience and, indeed, increasing the tescovine concentration up to 10%, the internal cohesion decreased and the product structure became easier to deform, while retaining its elasticity with significant reductions corresponding to the highest grape pomace integration (Table 3).

Gummosity and chewability displayed increasing trends from the untreated control to the 10% grape by-product addition, up to 6.2 and 6.4 times, respectively (Table 3), both just after the product manufacture and seven days later, indicating a denser and stickier texture, which implies a higher chewing time.

Natur Tanya (CTNT) tescovine powder addition led to the highest average values of all the textural parameters examined (Table 3 and Table 4).

The outcome of this study confirms that the rising addition of a solid ingredient into the sponge cakes influenced the texture of the final food, particularly increasing its hardness, with optimal tescovine powder concentrations of 3% to keep the product soft, consistently with the results of Pasukamonset et al. [35] and Nakov et. al. [29]. The former authors [35] reported the beneficial impact of integrating the powder of *Ciltoria ternatea* (CTE), an anthocyanin-rich plant [52], at 4 different concentrations (5%, 10%, 15% and 20% CTE), into the pastry product bread dip, which showed firmness increase upon rising powder addition. Opposite trend was recorded by Sergiacomo et al. [53], who detected the decrease of sponge cake texture upon augmenting addition of oat seeds to wheat flour, but only the 72-h sprouted seeds led to higher values than the untreated control. In our study, contrary to the texture outcome, reduced values were recorded of cohesiveness, which is the resistance of food material to deformation or compression between teeth before decomposition [54], elasticity, expressed by the level of a product strength following two compressions [55], and resilience, the ability of food to bounce back after the first compression. The mentioned trends suggest that the addition of a new ingredient in the original recipe leads to a decrease in internal cohesiveness as well as in the sample elasticity.

In previous research [56], the highest levels of elasticity and porosity in pastries were recorded in the untreated control, with drops by 5.9 and 19.9% of elasticity, and by 7.8 and 9.0% of porosity in cakes and rolls, respectively, upon 25% grape by-product addition. The increasing integration of the mentioned waste enhanced the spread ratio (SR) in cookies, like under non-cereal flour insertion into dough, supposedly related to gluten network reduction and fiber rise [29].

A texture drop caused by the amylolytic activity under 5% to 15% lingonberry integration was recorded in another study [57], with the 10–15% addition of lingonberry fruit into spelt flour leading to the optimal product; higher percentages resulted in less stable and harder to process dough, with lower attractiveness of pastry volume and appearance. The latter showed significant moisture decrease in other investigations, upon increasing grape pomace integration [29,56,58]. Rising protein trends were reported in bakery products added with *V. vinifera* by-products [56,59,60] or mushroom powder [61], but opposite reaction was recorded by other authors [29].

Pasukamonset et al. [35] also recorded higher values of gummosity, the force required to chew food until it is swallowed, and chewability, the energy quantity needed to disintegrate an aliment for swallowing [55], compared to the untreated control.

Nakov et al. [29] found that increasing incorporation of tescovine powder (applied at 4%, 6%, 8% and 10%), into pastry products enhanced the cake hardness, at the same time augmenting the chewability up to the 10% integration.

### 3.2. Quality Parameters, Water Activity and Color Components

The incorporation of tescovine powder into the sponge cake manufacturing remarkably influenced the quality properties of the finished products (Table 4).

Indeed, dry matter content and acidity significantly increased from the untreated control to the top grape pomace integration, up to 1.11 and 9.1 times, respectively, both just after production and seven days later. Similar trends were recorded for sugars, mineral substances and ash, whereas proteins and fats were not significantly affected by tescovine addition, despite a tendential decreasing trend with rising by-product powder percentage.

Referring to the 3 levels (3%, 5% and 10%) of grape pomace integrations into the sponge cakes, the average percentages of retention of the substances added in the finished product, are: 148%, 89%, 95%, 100% and 200% for sugars, fiber, proteins, fats and mineral substances, respectively.

Natur Tanya grape pomace powder integration elicited, on average, the highest values of sugars, acidity, mineral substances and ash, but the lowest pH (Table 5).

The pH values were gradually reduced by increasing the tescovine percentage into cakes, due to the organic acids in the *V. vinifera* by-product [20]. The untreated control had a pH of 7.63, typical of cheddar products [62], while values of 5.29 and 3.86 were recorded under the 10% tescovine addition, indicating the product acidification. The 3% tescovine integration into the cake did not significantly differ from the untreated control and, therefore, can be considered optimal. Nakov et al. [29] and Tolve et al. [31] recorded a pH decrease down to 5.4 and 4.0, respectively, in tescovine-enriched bakery products, as tescovine is a waste containing organic acids [12]. The moisture content also decreased in the fortified samples, according to Tseng and Zhao [63], due to the reduced hygroscopic effect and increased fiber content upon tescovine powder integration [64].

**Figure 2:**
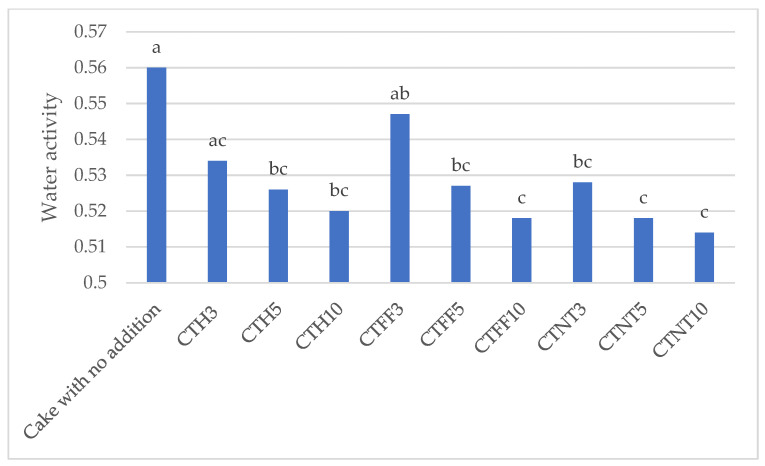
Water activity of cake added with grape pomace. Mean values followed by different letters are significantly different at *p* ≤ 0.05, according to Duncan test; n (number of replicates) = 3.

In the present research, after 7 days of storage under refrigeration (2–4 °C) in closed plastic boxes, the sponge cakes associated with the untreated control and to the lowest addition percentage of the three red grape pomace powders showed the highest water activity (Aw) values (0.56 in the control, and from 0.528 in CTNT3 to 0.547 in CTFF3), as can be observed in Figure 2. The by-product integration increase caused an Aw decrease, reaching values between 0.514 (CTNT10) and 0.520 (CTH10) under the 10% incorporation.

Water activity (Aw) levels for food products range between 0 and 1, being lower when the binding forces are stronger, and the lower the water content of the sample, the lower its water activity [65]. Water activity values below 0.6–0.7 (depending on the storage temperature or product pH) are considered to ensure optimal preservation [65]: Microorganism growth is inhibited at Aw levels < 0.91 (moisture below 30%) for most bacteria, <0.88 (moisture below 25%) for yeasts and <0.8 (moisture below 10%) for molds. When Aw values are between 0.5 and 0.6, water is found in the form of polymolecular layers, partially covering the surface of the substrate. The Aw greater than 0.6 corresponds to free water in liquid state, also called biological water, retained on the surface of the dehydrated layer only by capillary forces, which can be used by microorganisms for enzymatic reactions [65].

Grape pomace is a by-product rich in anthocyanins [66] which significantly contribute to the color profile modification of the final product. In this respect, the color component L* (lightness) in both the cake crust and the crumb showed lower values (Table 5), compared to the untreated control, giving the product a darker shade with purplish tinges. The natural pigments in tescovine composition [67] also led to the modification of the color components a* (green–red) and b* (yellow–blue).

The color component a* increased upon tescovine powder addition in the cake crumb, whereas the crust showed opposite trend, consistently with the reports of Tolve et al. [31] both in the bread crust and crumb added with grape pomace powder. Simonato et al. [68] stated that the color difference between crust and crumb may be due to the lower influence of high baking temperature on anthocyanins (responsible for tesccovine red color) in the crumb, compared to the crust.

The color component b* showed the same decreasing trend in both crust and crumb. The control sample values fell in the warm chromatic range, with golden-yellow hues, but the values significantly dropped upon the addition of tescovine powder, indicating a bluish color. Previously, Troilo et al. [60] reported that the values of the color component b* followed the same trend as our results; indeed, the analyzed muffins, which initially exhibited yellow hues, significantly decreased under the addition of grape powder, leading to a color change towards bluish. The color of the crust and crumb may closely relate to the Maillard reaction involving reducing sugars remarkably present in grape tescovine [29].

### 3.3. Antioxidant Compounds and Activity

The rising addition of grape pomace to the sponge cake enhanced the bioactive status, with the highest increase under the 10% Fiber Foods powder addition, by 592%, 190% and 556%, for the antioxidant activity, polyphenols and flavonoids, respectively (Table 6). The Fiber Foods integration did not significantly differ from Nature Tanya in the case of vitamin C and from Herbal Sana in the case of anthocyanins, and both mentioned antioxidants were not detected in the untreated control.

Referring to the 3 levels (3%, 5% and 10%) of grape pomace integrations into the sponge cakes, the average percentages of retention of the antioxidant compounds added in the finished product, are: 84%, 83%, 48% and 100% for vitamin C, polyphenols, flavonoids and anthocyanins, respectively; the antioxidant activity measured in the final cakes was the same as the theoretical one.

The addition of Fiber Foods tescovine powder resulted in the highest average values of the antioxidant activity and compounds measured, except for vitamin C which was best influenced by Natur Tanya (Table 6).

In previous research, the antioxidant activity was enhanced, compared to the untreated control, by the increasing integration of grape pomace in pastries up to 20% [60], 25% [56], or 30% [58,69], whereas the total flavonoid content augmented until the *V. vinifera* addition by 10% [69] or 25% [56]. Rising levels of antioxidant activity and flavonoids were recorded in pastries up to 25% lingonberry powder fortification [57].

The increasing integration of parsley leaves up to 4% [70] or grape pomace [56,71,72,73,74] into pastries enhanced phenolic compounds and antioxidant activity, compared to control. Total polyphenolic content also augmented in cookies upon powder integration of pomegranate rind [59], date seed [75], and flavonoids upon pumpkin peel, flesh, and seed addition [76].

However, other authors reported that: the interactions between phenolic compounds and food matrix proteins contribute to reducing the antioxidant properties in bakery products, lowering the fortification efficiency [70]; the abundance of flavonoids and polyphenols introduced by the added material inhibited the antioxidant activity in pastries [57].

The findings of the present study agree with the literature reports about the importance of grape skin as a rich anthocyanin source [77]. Martins et al. [78] found that the differences in antioxidant content between the experimental treatments relate to grape variety and the manufacturing process originating the by-product powder.

Alshawi et al. [79] recorded polyphenol augmentation in snacks upon the integration of 4 tescovine powder concentrations (5%, 10%, 15%, 20%), from 68.2 mg 100 g^−1^ in the untreated control to 214 mg 100 g^−1^ in the 20% tescovine added samples. Similar trends were found by Rainero et al. [32] upon red grape pomace additions to grisine-type product, with the total polyphenol content ranging from 72.2 mg GAE 100 g^−1^ DM to 171.8 mg GAE 100 g^−1^ DM.

Maner et al. [71] created a functional sweet product, integrating tescovine powder into a cookie, with healthy beneficial properties derived from the bioactive compounds present in the mentioned by-product [80], showing similar values of bioactive compounds to those recorded in our study. Other authors [70] reported the anthocyanin content increase to 3.512 mg g^−1^ in pastry products, compared to 0.163 mg g^−1^ in the untreated control, elicited by the integration of grape skin as a rich anthocyanin, hydroxcinnamic acids and flavonolic glycosides source. Similar increasing trends were observed in the final manufactured cookies: polyphenol content, from 0.05 to 0.46 mg g^−1^; antioxidant activity, from 11.7 to 76 mg g^−1^; flavonoid content, from 0.032 to 1.133 mg g^−1^ [70].

### 3.4. Sensory Characteristics

**Figure 3:**
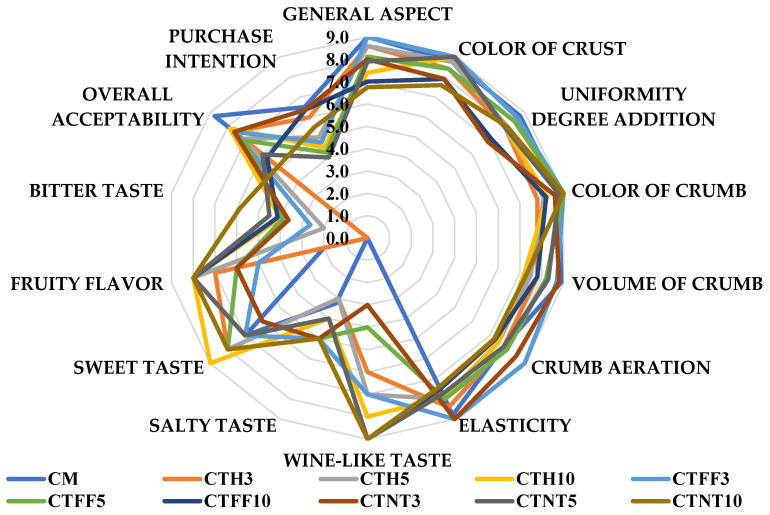
Sensory characteristics of sponge cake added with *Vitis Vinifera* pomace powder. CM—cake with no addition; CTH3%—cake with 3% Herbal Sana powder addition; CTH5%—cake with 5% Herbal Sana powder addition; CTH10%—cake with 10% Herbal Sana powder addition; CTFF3%—cake with 3% Fiber Foods powder addition; CTFF5%—cake with 5% Fiber Foods powder addition; CTFF10%—cake with 10% Fiber Foods powder addition; CTNT3%—cake with 3% Natur Tanya powder addition; CTNT5%—cake with 5% Natur Tanya powder addition; CTNT10%—cake with 10% Natur Tanya powder addition; n (number of replicates) = 3.

As shown in Figure 3, the sensory analysis revealed significant differences between the untreated control and the cakes added with red grape pomace powder. Most sensory characteristics decreased with rising powder addition from 3% to 10%, especially for the general aspect, crumb volume, aeration and elasticity. The color of the crust, crumb and the uniformity degree of the addition generally showed increasing trend. The wine-like, fruity flavor, sweet and bitter tastes also gradually increased, especially in the 10% tescovine added cakes. The highest overall acceptability was recorded for the untreated control, followed by the 3% grape pomace added pastries, and the purchase intention followed the same trend. The addition of 3% tescovine powder was fairly accepted by consumers.

The average influence of manufacturer tescovine powder on the sensory characteristics analyzed was controversial (Figure 3).

In previous studies, the best taste, flavor and overall acceptability were recorded in cookies upon the grape pomace addition of 10% [56,81,82] or 15% [71]; Nakov et al. [29] found the most appreciable nutritional value and sensory characteristics in cakes under the 4% grape powder integration and the most appropriate texture with the 6% insertion. In the mentioned investigations, appearance appreciation decreased upon 10% tescovine addition, because of sample darker color, and in another work the incorporation of 10% grape powder as a natural colorant of bakery food showed a significant influence on the product browned appearance [82].

In previous research [57], *Vaccinium vitis-idaea* fruit powder insertion into pastries determined the best taste/chewing, aroma, texture/porosity and general acceptance; the highest scores were attributed to 10% lingonberry integrated muffins and the lowest to the 25% addition. Furthermore, muffins enriched with 30% apple powder were the most appreciated by consumers due to their better taste, smell, mastication, and appearance [83].

## 4. Conclusions

From the present investigation, interesting perspectives have arisen regarding the creation of innovative functional food such as a sponge cake, combining wheat flour with grape pomace to improve the overall product properties under sustainable strategy. In this respect, both the tescovine manufacturer and the percentage of addition showed significant influence on the outcome obtained. Indeed, the fortification of sponge cakes by increasing integration of grape pomace from 3% to 10% enhanced some textural, quality and antioxidant properties of the manufactured pastries, though the color components and most sensory attributes were negatively affected. Natur Tanya tescovine powder addition was the most effective in enhancing the average values of the textural parameters examined, sugars, acidity, mineral substances, ash and vitamin C, whereas Fiber Foods grape pomace fostered at the best the antioxidant activity, polyphenols, flavonoids and anthocyanins. The influence of tescovine powder manufacturer on the sensory characteristics analyzed was controversial.

It can be inferred that the economically efficient valorization of grape waste by adding it to pastries can enhance the health properties of the bioactive compound enriched aliment in a sustainable way.

Future research should deepen the investigation of the benefits derived from pastry food integrated with beneficial by-product as well as their consumer acceptability under market conditions.

## Figures and Tables

**Table 1:** Chemical composition of grape pomaces integrated into sponge cake.

Tescovine Powder	pH	Sugars	Fiber	Pr	Fats	Min	AA	Vit C	Pol	Fl	Ant
	g 100 g^−1^						µmol Trolox Equivalents g^−1^		mg 100 g^−1^		
CTH	3.6	20	50.7	10.9	10.4	2.85	156	78	1120	33	17
CTFF	3.7	22	47.2	11.3	11.0	3.10	189	104	1160	35	19
CTNT	3.5	25	45.0	10.6	10.8	3.27	122	126	1060	29	15

CTH—Herbal Sana; CTFF Fiber Foods; CTNT Natur Tanya. Pr: Proteins; Min: Mineral substances; AA: Antioxidant activity; Vit C: Vitamin C; Fl: Flavonoids; Pol: Polyphenols; Ant: Anthocyanins. The quality and antioxidant compounds, and the antioxidant activity are expressed per fresh weight.

**Table 2:** Texture characteristics of cake added with grape pomace.

	Porosity (%)		k—Elasto-Plastic Coefficient		F Max Compression (N)	
Type of Grape Pomace × Percentage of Addition	Time 0	7 Days	Time 0	7 Days	Time 0	7 Days
Cake with no addition	60.4 a	53.0 ab	0.48 a	0.35 a	2.2 f	6.9 f
CTH3	59.6 ab	52.2 ab	0.37 b	0.26 b	2.2 f	6.1 f
CTH5	53.5 bc	51.0 ab	0.26 ce	0.23 c	3.6 e	9.7 e
CTH10	43.8 de	48.7 ac	0.22 ef	0.19 de	8.9 d	24.5 b
CTFF3	59.6 ab	55.7 a	0.28 cd	0.23 c	2.4 f	6.7 f
CTFF5	46.2 de	42.8 cd	0.25 de	0.19 de	3.7 e	8.9 e
CTFF10	42.8 e	40.0 d	0.18 f	0.13 f	9.6 c	18.0 d
CTNT3	59.0 ab	55.7 a	0.44 a	0.21 cd	3.5 e	9.0 e
CTNT5	55.4 ac	48.8 ac	0.37 b	0.18 e	10.6 b	20.8 c
CTNT10	50.0 cd	47.6 bc	0.32 bc	0.15 f	15.5 a	31.0 a

CTH3—cake with 3% Herbal Sana powder addition; CTH5—cake with 5% Herbal Sana powder addition; CTH10—cake with 10% Herbal Sana powder addition; CTFF3—cake with 3% Fiber Foods powder addition; CTFF5—cake with 5% Fiber Foods powder addition; CTFF10—cake with 10% Fiber Foods powder addition; CTNT3—cake with 3% Natur Tanya powder addition; CTNT5—cake with 5% Natur Tanya powder addition; CTNT10—cake with 10% Natur Tanya powder addition. Within each column, mean values followed by different letters are significantly different at *p* ≤ 0.05, according to Duncan test; n (number of replicates) = 3.

**Table 3:** Texture characteristics of cake added with grape pomace.

	Resilience		Cohesiveness (-)		Elasticity (-)		Gummosity (N)		Chewability (N)	
Type of Grape Pomace × Percentage of Addition	Time 0	After 7 Days	Time 0	After 7 Days	Time 0	After 7 Days	Time 0	After 7 Days	Time 0	After 7 Days
Cake with no addition	0.75 a	0.06 f	0.83 ab	0.70 a	2.87 ab	2.57 ac	1.8 e	3.3 e	4.7 g	7.4 h
CTH3	0.70 ab	0.27 d	0.81 ab	0.59 b	2.80 ac	2.58 ab	1.8 e	4.6 d	5.2 fg	9.3 g
CTH5	0.69 ab	0.32 c	0.73 c	0.51 cd	2.79 bc	2.47 cd	2.8 d	4.9 d	7.5 e	12.2 e
CTH10	0.63 ab	0.47 b	0.71 c	0.46 e	2.66 c	2.23 e	6.5 c	11.0 b	18.1 c	24.4 c
CTFF3	0.69 ab	0.12 e	0.82 ab	0.48 de	2.97 a	2.60 ab	1.9 e	2.8 e	5.7 f	7.3 h
CTFF5	0.66 ab	0.32 c	0.81 ab	0.38 f	2.87 ab	2.50 bd	3.1 d	3.3 e	8.8 d	10.7 f
CTFF10	0.57 b	0.60 a	0.70 c	0.34 g	2.47 d	2.45 d	6.8 c	8.2 c	17.9 c	19.6 d
CTNT3	0.78 a	0.31 cd	0.85 a	0.54 c	2.88 ab	2.65 a	2.7 d	4.6 d	8.0 de	12.3 e
CTNT5	0.71 ab	0.32 c	0.78 b	0.53 c	2.87 ab	2.46 d	8.2 b	11.3 b	22.8 b	27.7 b
CTNT10	0.58 b	0.47 b	0.71 c	0.27 h	2.70 c	2.27 e	11.1 a	14.5 a	30.3 a	33.0 a

CTH3—cake with 3% Herbal Sana powder addition; CTH5—cake with 5% Herbal Sana powder addition; CTH10—cake with 10% Herbal Sana powder addition; CTFF3—cake with 3% Fiber Foods powder addition; CTFF5—cake with 5% Fiber Foods powder addition; CTFF10—cake with 10% Fiber Foods powder addition; CTNT3—cake with 3% Natur Tanya powder addition; CTNT5—cake with 5% Natur Tanya powder addition; CTNT10—cake with 10% Natur Tanya powder addition. Within each column, mean values followed by different letters are significantly different at *p* ≤ 0.05, according to Duncan test; n (number of replicates) = 3.

**Table 4:** Quality characteristics of cake added with grape pomace.

Type of Grape Pomace × Percentage of Addition	Dry Matter g 100 g^−1^		Sugars g 100 g^−1^	Fiber g 100 g^−1^	Acidity g 100 g^−1^		pH		Proteins g 100 g^−1^	Fats g 100 g^−1^	Mineral Substances g 100 g^−1^	Ash g 100 g^−1^
	Time 0	After 7 Days			Time 0	After 7 Days	Time 0	After 7 Days				
Cake with no addition	64.2 e	65.0 g	4.4 f	2.30 g	0.18 h	0.19 g	7.63 a	7.63 a	8.6	12.9	1.79 e	0.07 e
CTH3	65.4 d	66.4 e	5.5 e	3.54 ef	0.51 g	0.57 f	6.10 c	6.35 c	8.4	12.8	2.28 de	0.09 de
CTH5	66.5 c	67.1 d	6.0 de	4.05 cd	0.61 f	0.65 f	5.69 d	5.84 e	8.3	12.7	3.34 c	0.09 de
CTH10	69.3 b	71.2 b	11.4 c	6.12 a	0.81 e	1.20 cd	4.65 g	4.93 h	8.1	12.7	5.01 a	0.17 b
CTFF3	65.6 d	66.1 e	4.5 f	3.56 ef	0.84 e	0.87 e	6.57 b	6.62 b	8.5	12.8	2.52 d	0.09 de
CTFF5	66.5 c	67.2 d	5.4 e	4.37 c	1.08 d	1.08 d	6.09 c	6.24 d	8.4	12.8	3.10 c	0.10 d
CTFF10	71.5 a	72.0 a	6.6 d	5.53 b	1.27 c	1.53 b	5.30 f	5.34 g	8.1	12.6	4.97 a	0.13 c
CTNT3	64.6 e	65.7 f	6.4 d	3.44 f	1.19 c	1.33 c	5.54 e	5.77 f	8.4	12.7	3.05 c	0.09 de
CTNT5	69.8 b	70.2 c	14.0 b	3.88 de	1.41 b	1.67 ab	4.15 h	4.97 h	8.2	12.7	4.31 b	0.13 c
CTNT10	71.3 a	72.3 a	17.0 a	5.86 ab	1.64 a	1.73 a	3.90 i	4.23 i	8.1	12.6	4.84 a	0.26 a
									n.s.	n.s.		

The values of all the parameters, except pH, are expressed on fresh weight basis. CTH3—cake with 3% Herbal Sana powder addition; CTH5—cake with 5% Herbal Sana powder addition; CTH10—cake with 10% Herbal Sana powder addition; CTFF3—cake with 3% Fiber Foods powder addition; CTFF5—cake with 5% Fiber Foods powder addition; CTFF10—cake with 10% Fiber Foods powder addition; CTNT3—cake with 3% Natur Tanya powder addition; CTNT5—cake with 5% Natur Tanya powder addition; CTNT10—cake with 10% Natur Tanya powder addition. Within each column, mean values followed by different letters are significantly different at *p* ≤ 0.05, according to Duncan test; n (number of replicates) = 3. n.s. not significant.

**Table 5:** Color characteristics of cake added with grape pomace.

	L*		a*		b*	
Type of Grape Pomace × Percentage of Addition	In Crust	In Crumb	In Crust	In Crumb	In Crust	In Crumb
Cake with no addition	57.7 a	76.6 a	19.0 a	4.5 h	36.9 a	42.1 a
CTH3	49.4 b	45.4 b	8.5 e	6.2 g	20.3 b	11.2 cd
CTH5	37.4 e	37.9 e	7.6 f	6.6 f	13.0 e	8.0 g
CTH10	30.7 hi	33.3 d	6.4 g	7.5 cd	7.4 h	5.3 i
CTFF3	41.4 d	43.0 d	12.6 b	9.9 b	18.3 c	13.6 b
CTFF5	33.3 f	36.8 f	9.8 c	10.1 b	10.0 f	10.8 d
CTFF10	31.7 gh	35.0 g	9.1 d	11.0 a	8.2 gh	10.0 e
CTNT3	44.0 c	44.3 c	9.2 d	7.1 e	16.9 d	11.6 c
CTNT5	32.6 fg	38.0 e	7.5 f	7.6 d	8.7 g	9.0 f
CTNT10	29.7 i	33.5 h	5.1 h	7.8 c	3.9 i	6.5 h

CTH3—cake with 3% Herbal Sana powder addition; CTH5—cake with 5% Herbal Sana powder addition; CTH10—cake with 10% Herbal Sana powder addition; CTFF3—cake with 3% Fiber Foods powder addition; CTFF5—cake with 5% Fiber Foods powder addition; CTFF10—cake with 10% Fiber Foods powder addition; CTNT3—cake with 3% Natur Tanya powder addition; CTNT5—cake with 5% Natur Tanya powder addition; CTNT10—cake with 10% Natur Tanya powder addition. Within each column, mean values followed by different letters are significantly different at *p* ≤ 0.05, according to Duncan test; n (number of replicates) = 3.

**Table 6:** Antioxidant characteristics of cake added with grape pomace.

Type of Grape Pomace × Percentage of Addition	Antioxidant Activity (µmol Trolox Equivalents g^−1^)	Vitamin C (mg 100 g^−1^)	Polyphenols (mg GAE g^−1^)	Flavonoids (mg CE g^−1^)	Anthocyanins (mg g^−1^)
Cake with no addition	3.8 g	0.0 g	1.83 d	0.27 h	0.00 g
CTH3	9.8 e	1.8 f	1.36 e	0.77 f	0.07 e
CTH5	12.4 d	3.5 e	1.86 d	1.07 e	0.11 bc
CTH10	15.7 b	5.3 c	2.75 b	1.41 b	0.15 a
CTFF3	14.1 c	3.5 e	1.42 e	1.14 d	0.09 d
CTFF5	16.0 b	4.4 d	2.29 c	1.27 c	0.12 b
CTFF10	22.5 a	8.8 a	3.48 a	1.50 a	0.16 a
CTNT3	7.7 f	4.1 d	1.06 f	0.50 g	0.04 f
CTNT5	8.0 f	6.3 b	1.81 d	0.75 f	0.07 e
CTNT10	10.0 e	8.8 a	1.89 d	1.08 e	0.10 cd

The values of all the parameters are expressed on dry weight basis, except for vitamin C on fresh weight basis. CTH3—cake with 3% Herbal Sana powder addition; CTH5—cake with 5% Herbal Sana powder addition; CTH10—cake with 10% Herbal Sana powder addition; CTFF3—cake with 3% Fiber Foods powder addition; CTFF5—cake with 5% Fiber Foods powder addition; CTFF10—cake with 10% Fiber Foods powder addition; CTNT3—cake with 3% Natur Tanya powder addition; CTNT5—cake with 5% Natur Tanya powder addition; CTNT10—cake with 10% Natur Tanya powder addition. Within each column, mean values followed by different letters are significantly different at *p* ≤ 0.05, according to Duncan test; n (number of replicates) = 3.

## Data Availability

Data are available upon request to the corresponding authors.
